# Correction to: Strong Positive Selection in *Aedes aegypti* and the Rapid Evolution of Insecticide Resistance

**DOI:** 10.1093/molbev/msag024

**Published:** 2026-02-09

This is a correction to: R Rebecca Love, Josh R Sikder, Rafael J Vivero, Daniel R Matute, Daniel R Schrider, Strong Positive Selection in *Aedes aegypti* and the Rapid Evolution of Insecticide Resistance, *Molecular Biology and Evolution*, Volume 40, Issue 4, April 2023, https://doi.org/10.1093/molbev/msad072

In section **Materials & Methods**, under heading **Sample Collection**, the penultimate and previous sentences of the second paragraph, text ahouls read: “All specimen collections were performed following the guidelines of permits No 001566 and 002662.” instead of: “All specimen collections were performed following the guidelines of the Colombian decree nNo. 1376. No specific permits were required for this study.”

This is outlined only in this correction notice to preserve the version of record.

