# Supplementary material for: Strong Positive Selection in Aedes aegypti and the Rapid Evolution of Insecticide Resistance
Source: Mol Biol Evol. 2023 Mar 27;40(4):msad072. doi: 10.1093/molbev/msad072 (PMC10118305; doi:10.1093/molbev/msad072)
Supplement: msad072_Supplementary_Data [file msad072_supplementary_data.zip › Love_et_al_0643_supplemental_tables.pdf]

SUPPLEMENTAL TABLES

Table S1. Specimen provenances (separate file).

Table S2. Alignment quality by country.

	Mean (median) average read depth, per specimen	mean (median) % reads mapping, per specimen
Brazil	13.06 (13.08)	97.82 (97.89)
Colombia	21.19 (20.90)	96.69 (98.37)
USA	10.16 (9.03)	98.19 (98.19)
Gabon	14.82 (14.94)	94.80 (94.65)
Kenya	15.64 (14.79)	96.73 (96.82)
Senegal	16.42 (15.80)	96.94 (97.00)

Table S3. Total and mean number of variants segregating in each cohort after removal of close kin. Columns normalized by cohort sample size have been rounded to the nearest SNP.

	Total unfiltered SNP calls, before kin removal	Unfiltered SNP calls, per specimen, before kin removal	Total filtered SNP calls	Filtered SNP calls, per specimen	Total SNP calls after kin removal	Per specimen
Brazil	54,671,215	3,037,290	16,437,896	913,216	16,086,055	1,005,378
Colombia	71,992,561	2,117,428	20,906,880	614,908	20,726,790	647,712
USA	75,753,820	2,805,697	23,762,218	880,082	23,624,027	908,616
Gabon	99,034,712	7,618,055	32,975,726	2,536,594	32,975,726	2,536,594
Kenya	132,966,260	6,998,224	45,700,795	2,405,305	44,493,977	2,617,293
Senegal	106,893,209	5,344,660	34,151,512	1,707,576	33,792,786	1,778,568

Table S4. Nucleotide diversity and Tajima's D in six countries.

Country	Mean (median) nucleotide diversity	Mean (median) Tajima's D
Brazil	0.0054 (0.0053)	0.803 (0.841)
Colombia	0.0054 (0.0053)	0.407 (0.459)
USA	0.0067 (0.0064)	0.463 (0.499)
Gabon	0.0097 (0.0099)	0.041 (0.037)
Kenya	0.0097 (0.0099)	-0.837 (-0.845)
Senegal	0.0080 (0.0081)	-0.528 (-0.524)

Table S5. Mean (median) F_{ST} calculated between the six countries in our combined dataset.

	Brazil	Colombia	USA	Gabon	Kenya	Senegal
Brazil						
Colombia	0.084 (0.083)					
USA	0.094 (0.094)	0.101 (0.099)				
Gabon	0.250 (0.252)	0.276 (0.279)	0.203 (0.206)			
Kenya	0.206 (0.203)	0.232 (0.232)	0.166 (0.174)	0.067 (0.060)		
Senegal	0.145 (0.144)	0.161 (0.160)	0.111 (0.112)	0.094 (0.082)	0.108 (0.104)	

Table S6. Mean (median) linkage disequilibrium by country across all three chromosomes, calculated in 500 kb nonoverlapping windows.

Population	Mean (median) r^2
Brazil	0.038 (0.036)
Colombia	0.015 (0.015)
USA	0.015 (0.015)
Gabon	0.022 (0.022)
Kenya	0.011 (0.008)
Senegal	0.016 (0.015)

Table S7. Genes inside selected putative sweep regions (separate file).

Table S8. Distribution of outlier windows for G123 and G2/G1 in two candidate sweep regions (separate file).

Table S9. Linkage disequilibrium (r^2) between five known insecticide resistance loci, in the USA cohort.

	F1534C (315939224)	V1016I (315983763)	I915K (315999297)	S723T (316014588)	V410L (316080722)
F1534C (315939224)					
V1016I (315983763)	0.358143				
I915K (315999297)	0.034192	0.414925			
S723T (316014588)	0.218757	0.568166	0.291030		
V410L (316080722)	0.012860	0.368465	0.390857	0.739800	
