# Supplementary material for: Strong Positive Selection in Aedes aegypti and the Rapid Evolution of Insecticide Resistance
Source: Mol Biol Evol. 2023 Mar 27;40(4):msad072. doi: 10.1093/molbev/msad072 (PMC10118305; doi:10.1093/molbev/msad072)
Supplement: msad072_Supplementary_Data [file msad072_supplementary_data.zip › Love_et_al_6043_supplemental_figures_compiled.pdf]

SUPPLEMENTAL FIGURE LEGENDS

Figure S1. Kinship coefficients between all 131 specimens.

Figure S2. Percent variance of the genome-wide PCA explained by each of the first ten principal components.

Figure S3. Distributions of nucleotide diversity π and Tajima's *D* by chromosome and country.

Figure S4. Nucleotide diversity π and Tajima's *D* along each chromosome, by country.

Figure S5. Distribution of linkage disequilibrium (r^2) by chromosome and country.

Figure S6. Site frequency spectra for each country.

Figure S7. Composite likelihood ratio (CLR) as calculated by SweepFinder2, and nearby genes, for selected candidate sweeps. In each subfigure, the colored dots at the top of the main panel indicate individual test loci at 1 kb intervals that have sweep likelihood (CLR) values at or above the 99th percentile for that country. Main panel line plots show CLR values averaged in 10 kb non-overlapping windows. Rectangles below the main panel indicate genes. Protein-coding genes with a predicted function are shown as white rectangles with black diagonal hatch marks in the upper half of the panel; all other genes are shown as transparent grey rectangles in the

lower half of the panel. In subfigure B, the blue rectangles with the “x” hatch marks in the upper half of the panel mark glutathione transferase genes. In subfigure E, the blue rectangle with the “x” hatch marks in the lower half of the panel marks the voltage-sensitive sodium channel gene *Vgsc* (AAEL023266).

Figure S8. Functional enrichment of genes overlapping outlier sweep loci, across six cohorts. The left panel shows the result with genes only; in the right panel, a 1 kb buffer was added up- and downstream of each gene before calculating enrichment. A black box at the intersection of a country column and GO term row indicates that term is enriched in genes overlapping outlier windows in that cohort.

Figure S9. Three countries show outlier windows overlapping a cluster of histone genes (red) on chromosome 3. Each panel labeled with a cohort shows individual outlier windows between 340.5 and 342.5 Mb on chromosome 3. Elevated set of red rectangles below the main plot show histone genes; all other genes are represented by grey rectangles.

Figure S10. G123 and G2/G1 statistics calculated in the three cohorts from the Americas in the vicinity of two candidate sweeps occurring near or overlapping genes with a role in insecticide resistance. Genes are marked as in Figure S7. The left panel corresponds to candidate sweep “a” on Figure 2, and to Figure S7a; glutathione transferases are indicated in blue. The right panel corresponds to candidate sweep “b” on Figure 2, and to Figure S7b; *Vgsc* (AAEL023266) is indicated with blue cross-hatching.

Figure S11. Nucleotide diversity π and Tajima's *D* in the vicinity of *Vgsc* (AAEL023266) as shown in Figure 4 (calculated by country in 500 kb windows slid by 50 kb), presented as *z*-scores (number of standard deviations from the mean, as calculated across the entire genome).

Figure S12. Linkage disequilibrium r^2 between individual variants in the vicinity of *Vgsc* (AAEL023266) on AaegL5_3 for specimens from Brazil and Colombia (left panel), the USA (middle panel), and from all three of those countries (right panel).

Figure S13. Alignment depth and mapping quality on chromosome 3 from 310 to 320 Mb. Values were averaged across all specimens, and then averaged in 10 kb windows slid 1 kb. The five Xs at the bottom of the plot indicate the location of the five focal loci.

Figure S14. Ribbon plot showing mummer alignment between NIGP01000811, and chromosome 3. Alignments shorter than 1 kb or with less than 90% identity between query and subject are excluded.

Figure S15. Principal component analysis of *Vgsc* (AAEL023266) and surrounding region using genotypes called with bcftools. PCA are shown without (left) and with (right) masking genotypes with a genotype quality less than 20.

Figure S16. Nucleotide diversity π from 310-320 Mb on AaegL5_3 for Brazilian and Colombian specimens in the top and bottom clusters of the *Vgsc* PCA. Values for entire Brazil and Colombia cohorts shown as grey solid and grey dashed lines, respectively. The vertical

dashed lines mark the boundaries of *Vgsc* (315,926,360-316,405,639); the five Xs at the bottom indicate the locations of the five focal loci.

Figure S17. F_{ST} calculated between specimens from South America falling in the top and bottom clusters of the PCA shown in Figure 3, or in other words, between specimens with the haplotype carrying the resistant allele at all five focal loci and between specimens with the haplotype carrying the resistant allele at F1534C only. The top panel shows F_{ST} in the region from 310-320 Mb, calculated in 50 kb windows slid 5 kb; the middle panel shows F_{ST} in *Vgsc* and its immediate surroundings, calculated in 10 kb windows slid 1 kb. Dashed vertical lines show the boundaries of *Vgsc*. Orange dots in the middle panel represent F_{ST} between top and bottom clusters at individual variants. The bottom panel shows the mean number of variants found in each window when calculating F_{ST} (at the 10 kb scale; middle panel), measured on the left axis, and the mean percentage of variants deemed "accessible" (that is, not in a repetitive region or a region that does not map uniquely within the genome; see Methods) on the right axis. At the far bottom of the plot, *Vgsc* exons are indicated with black bars, and the locations of the five focal loci are marked with dotted vertical lines that continue into the bottom and middle panels.

Figure S18. Nucleotide diversity π from 310-320 Mb on AaegL5_3 for specimens from Brazil, Colombia, and California, with the latter subdivided into three genetic clusters as in Lee et al. 2019. The vertical dashed lines mark the boundaries of *Vgsc* (315,926,360-316,405,639); the five Xs at the bottom of the Tajima's *D* plot indicate the locations of the five focal loci.

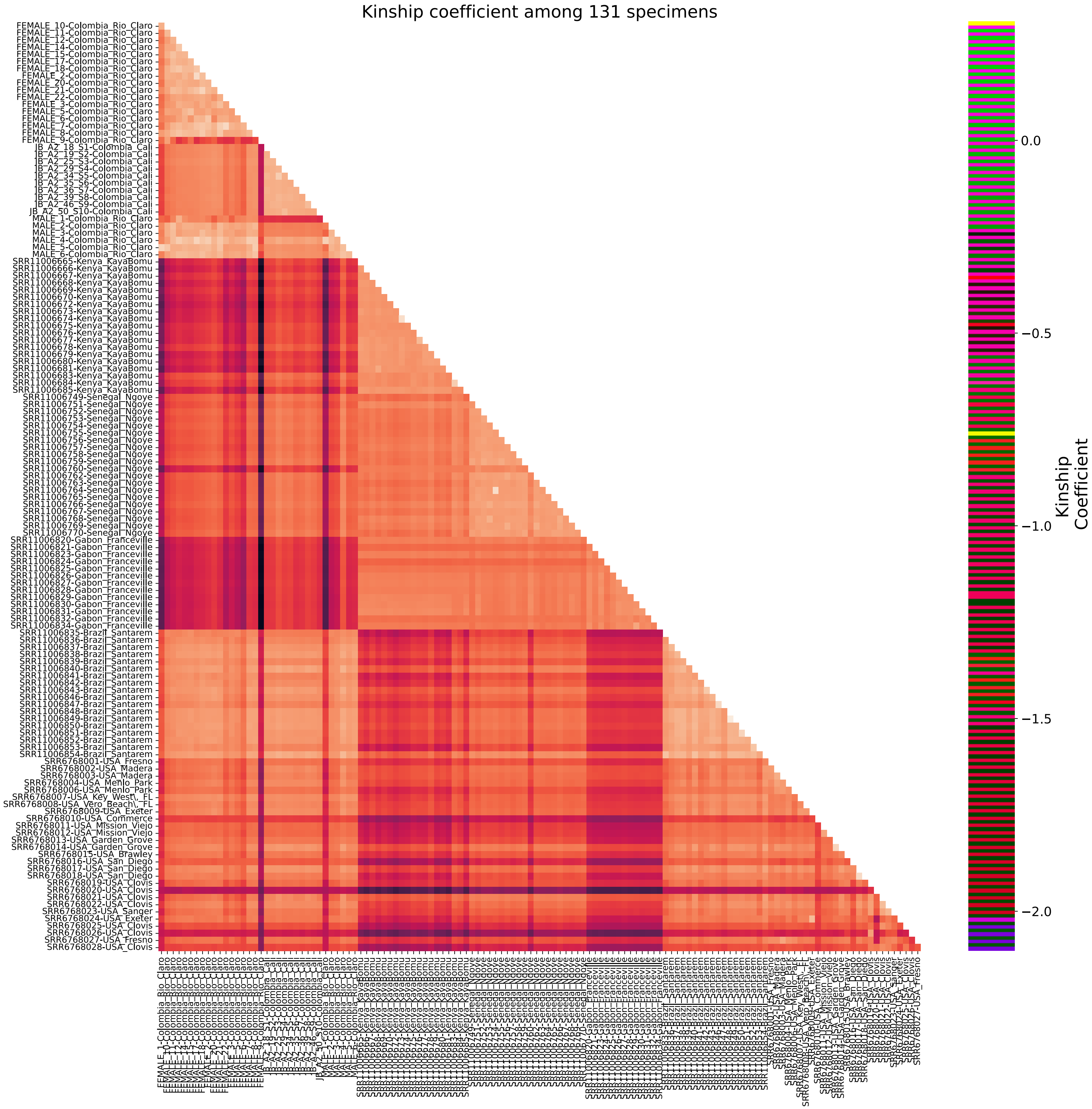

Kinship coefficient among 131 specimens
FEMALE_10-Colombia_Rio_Claro
FEMALE_11-Colombia_Rio_Claro
FEMALE_12-Colombia_Rio_Claro
FEMALE_14-Colombia_Rio_Claro
FEMALE_15-Colombia_Rio_Claro
FEMALE_17-Colombia_Rio_Claro
FEMALE_18-Colombia_Rio_Claro
FEMALE_2-Colombia_Rio_Claro
FEMALE_20-Colombia_Rio_Claro
FEMALE_21-Colombia_Rio_Claro
FEMALE_22-Colombia_Rio_Claro
FEMALE_3-Colombia_Rio_Claro
FEMALE_5-Colombia_Rio_Claro
FEMALE_6-Colombia_Rio_Claro
FEMALE_7-Colombia_Rio_Claro
FEMALE_8-Colombia_Rio_Claro
FEMALE_9-Colombia_Rio_Claro
JB_A2_18_S1-Colombia_Cali
JB_A2_19_S2-Colombia_Cali
JB_A2_25_S3-Colombia_Cali
JB_A2_29_S4-Colombia_Cali
JB_A2_34_S5-Colombia_Cali
JB_A2_35_S6-Colombia_Cali
JB_A2_36_S7-Colombia_Cali
JB_A2_39_S8-Colombia_Cali
JB_A2_46_S9-Colombia_Cali
JB_A2_50_S10-Colombia_Cali
MALE_1-Colombia_Rio_Claro
MALE_2-Colombia_Rio_Claro
MALE_3-Colombia_Rio_Claro
MALE_4-Colombia_Rio_Claro
MALE_5-Colombia_Rio_Claro
MALE_6-Colombia_Rio_Claro
SRR11006665-Kenya_KayaBomu
SRR11006666-Kenya_KayaBomu
SRR11006667-Kenya_KayaBomu
SRR11006668-Kenya_KayaBomu
SRR11006669-Kenya_KayaBomu
SRR11006670-Kenya_KayaBomu
SRR11006672-Kenya_KayaBomu
SRR11006673-Kenya_KayaBomu
SRR11006674-Kenya_KayaBomu
SRR11006675-Kenya_KayaBomu
SRR11006676-Kenya_KayaBomu
SRR11006677-Kenya_KayaBomu
SRR11006678-Kenya_KayaBomu
SRR11006679-Kenya_KayaBomu
SRR11006680-Kenya_KayaBomu
SRR11006681-Kenya_KayaBomu
SRR11006683-Kenya_KayaBomu
SRR11006684-Kenya_KayaBomu
SRR11006685-Kenya_KayaBomu
SRR11006749-Senegal_Ngoye
SRR11006751-Senegal_Ngoye
SRR11006752-Senegal_Ngoye
SRR11006753-Senegal_Ngoye
SRR11006754-Senegal_Ngoye
SRR11006755-Senegal_Ngoye
SRR11006756-Senegal_Ngoye
SRR11006757-Senegal_Ngoye
SRR11006758-Senegal_Ngoye
SRR11006759-Senegal_Ngoye
SRR11006760-Senegal_Ngoye
SRR11006762-Senegal_Ngoye
SRR11006763-Senegal_Ngoye
SRR11006764-Senegal_Ngoye
SRR11006765-Senegal_Ngoye
SRR11006766-Senegal_Ngoye
SRR11006767-Senegal_Ngoye
SRR11006768-Senegal_Ngoye
SRR11006769-Senegal_Ngoye
SRR11006770-Senegal_Ngoye
SRR11006820-Gabon_Franceville
SRR11006821-Gabon_Franceville
SRR11006823-Gabon_Franceville
SRR11006824-Gabon_Franceville
SRR11006825-Gabon_Franceville
SRR11006826-Gabon_Franceville
SRR11006827-Gabon_Franceville
SRR11006828-Gabon_Franceville
SRR11006829-Gabon_Franceville
SRR11006830-Gabon_Franceville
SRR11006831-Gabon_Franceville
SRR11006832-Gabon_Franceville
SRR11006834-Gabon_Franceville
SRR11006835-Brazil_Santarem
SRR11006836-Brazil_Santarem
SRR11006837-Brazil_Santarem
SRR11006838-Brazil_Santarem
SRR11006839-Brazil_Santarem
SRR11006840-Brazil_Santarem
SRR11006841-Brazil_Santarem
SRR11006842-Brazil_Santarem
SRR11006843-Brazil_Santarem
SRR11006846-Brazil_Santarem
SRR11006847-Brazil_Santarem
SRR11006848-Brazil_Santarem
SRR11006849-Brazil_Santarem
SRR11006850-Brazil_Santarem
SRR11006851-Brazil_Santarem
SRR11006852-Brazil_Santarem
SRR11006853-Brazil_Santarem
SRR11006854-Brazil_Santarem
SRR6768001-USA_Fresno
SRR6768002-USA_Madera
SRR6768003-USA_Madera
SRR6768004-USA_Menlo_Park
SRR6768006-USA_Menlo_Park
SRR6768007-USA_Key_West\,_FL
SRR6768008-USA_Vero_Beach\,_FL
SRR6768009-USA_Exeter
SRR6768010-USA_Commerce
SRR6768011-USA_Mission_Viejo
SRR6768012-USA_Mission_Viejo
SRR6768013-USA_Garden_Grove
SRR6768014-USA_Garden_Grove
SRR6768015-USA_Brawley
SRR6768016-USA_San_Diego
SRR6768017-USA_San_Diego
SRR6768018-USA_San_Diego
SRR6768019-USA_Clovis
SRR6768020-USA_Clovis
SRR6768021-USA_Clovis
SRR6768022-USA_Clovis
SRR6768023-USA_Sanger
SRR6768024-USA_Exeter
SRR6768025-USA_Clovis
SRR6768026-USA_Clovis
SRR6768027-USA_Fresno
SRR6768028-USA_Clovis
0.0
−0.5
−1.0
−1.5
−2.0
Kinship Coefficient

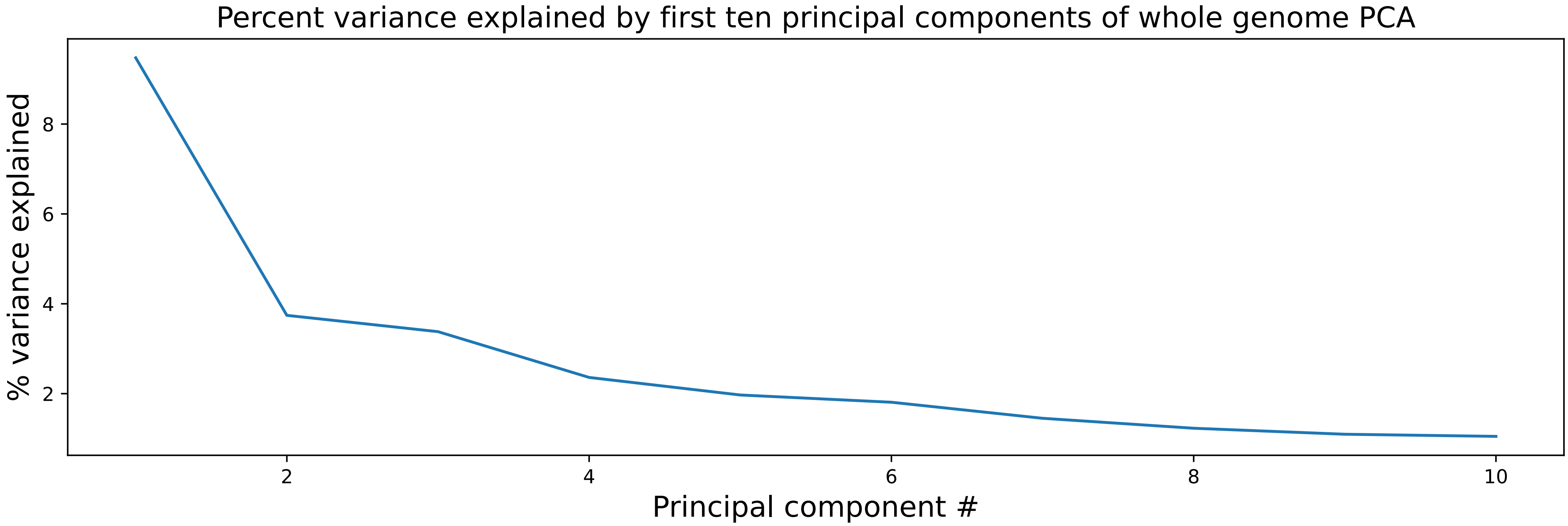
Percent variance explained by first ten principal components of whole genome PCA
% variance explained
8
6
4
2
2
4
6
8
10
Principal component #

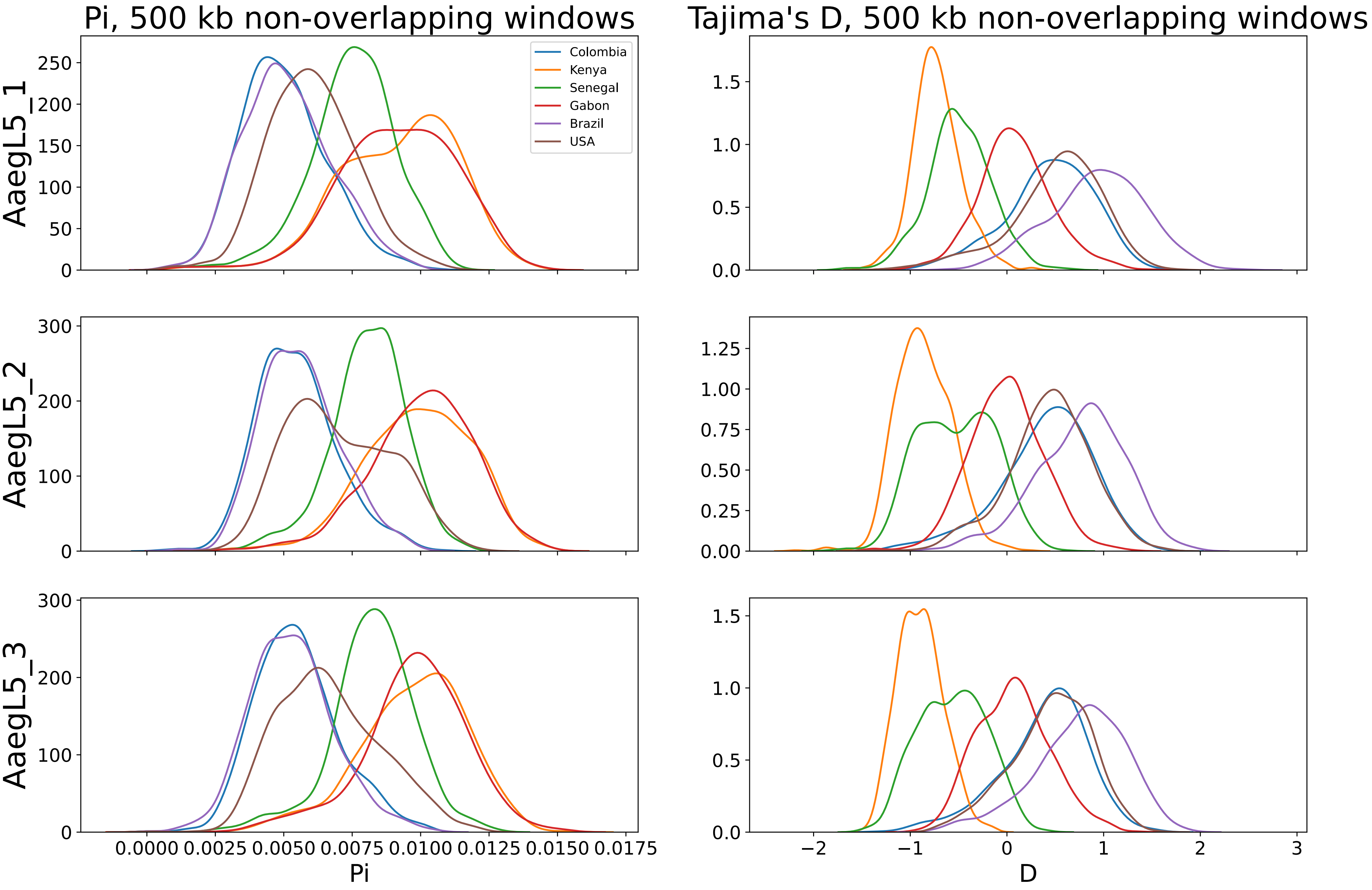
Pi, 500 kb non-overlapping windows
Tajima's D, 500 kb non-overlapping windows
AaegL5_1
AaegL5_2
AaegL5_3
Colombia
Kenya
Senegal
Gabon
Brazil
USA
Pi
D
0.0000 0.0025 0.0050 0.0075 0.0100 0.0125 0.0150 0.0175
−2 −1 0 1 2 3

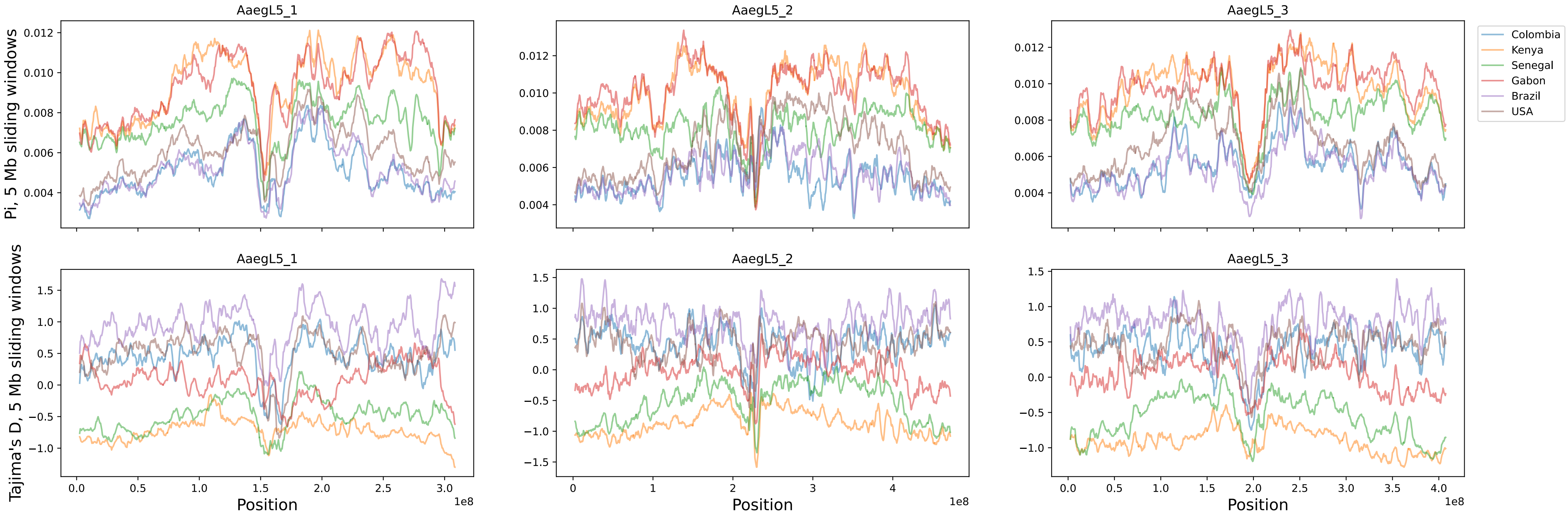

AaegL5_1
AaegL5_2
AaegL5_3
Pi, 5 Mb sliding windows
Tajima's D, 5 Mb sliding windows
Position
1e8
Colombia
Kenya
Senegal
Gabon
Brazil
USA

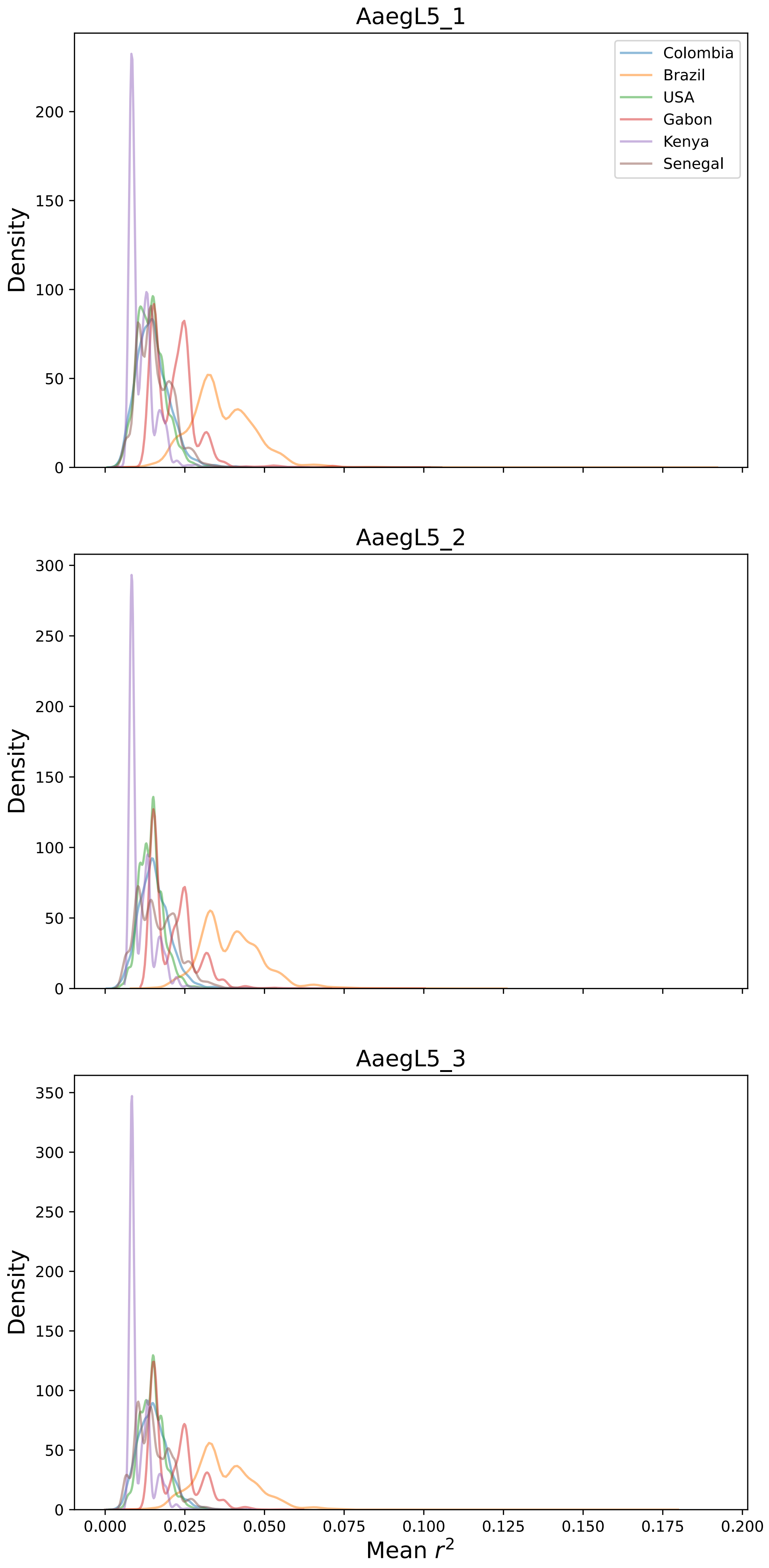

AaegL5_1
Colombia
Brazil
USA
Gabon
Kenya
Senegal
Density
AaegL5_2
Density
AaegL5_3
Density
0.000
0.025
0.050
0.075
0.100
0.125
0.150
0.175
0.200
Mean r^2

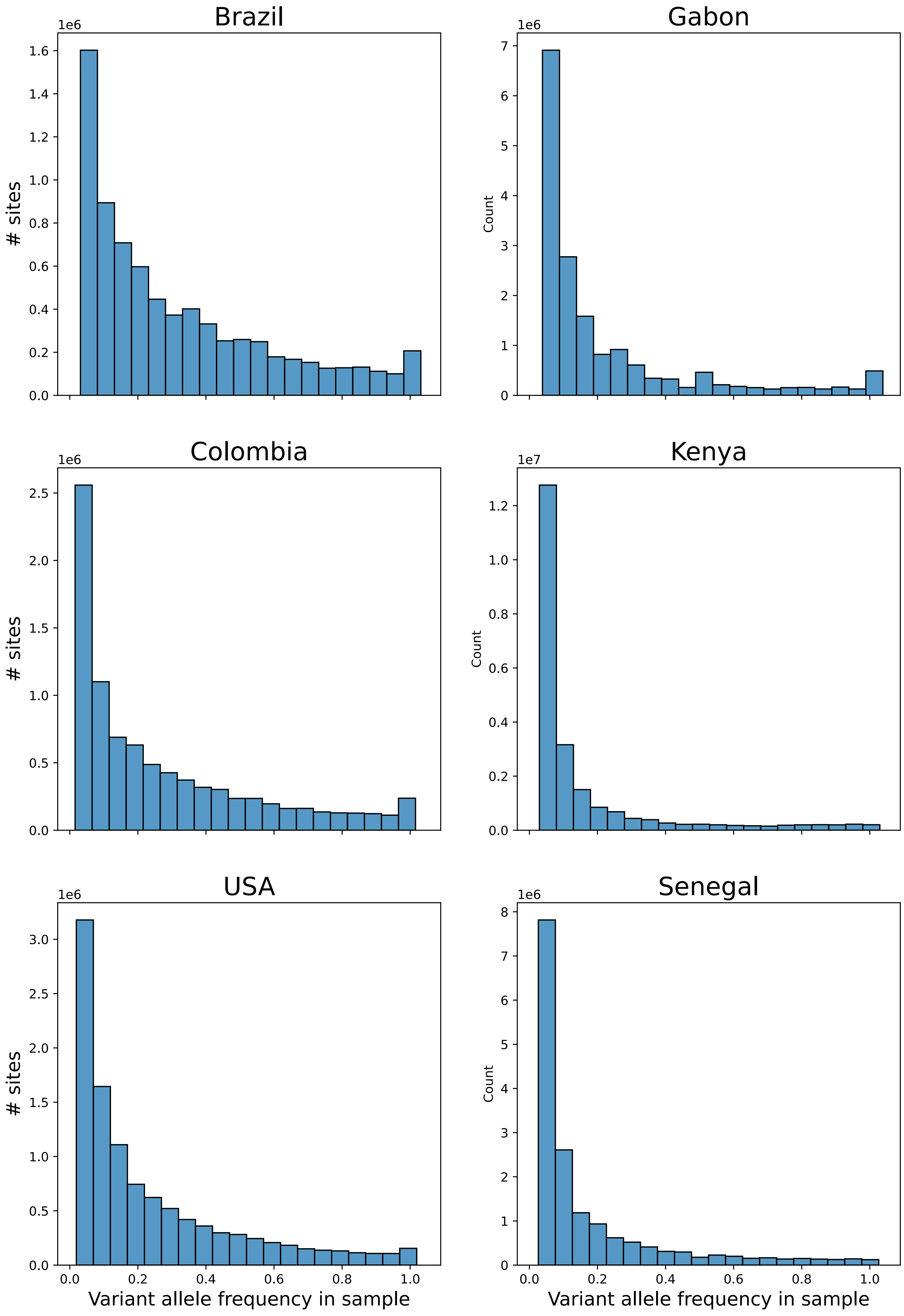

Brazil
Gabon
Colombia
Kenya
USA
Senegal
sites
Count
1e6
1e7
Variant allele frequency in sample

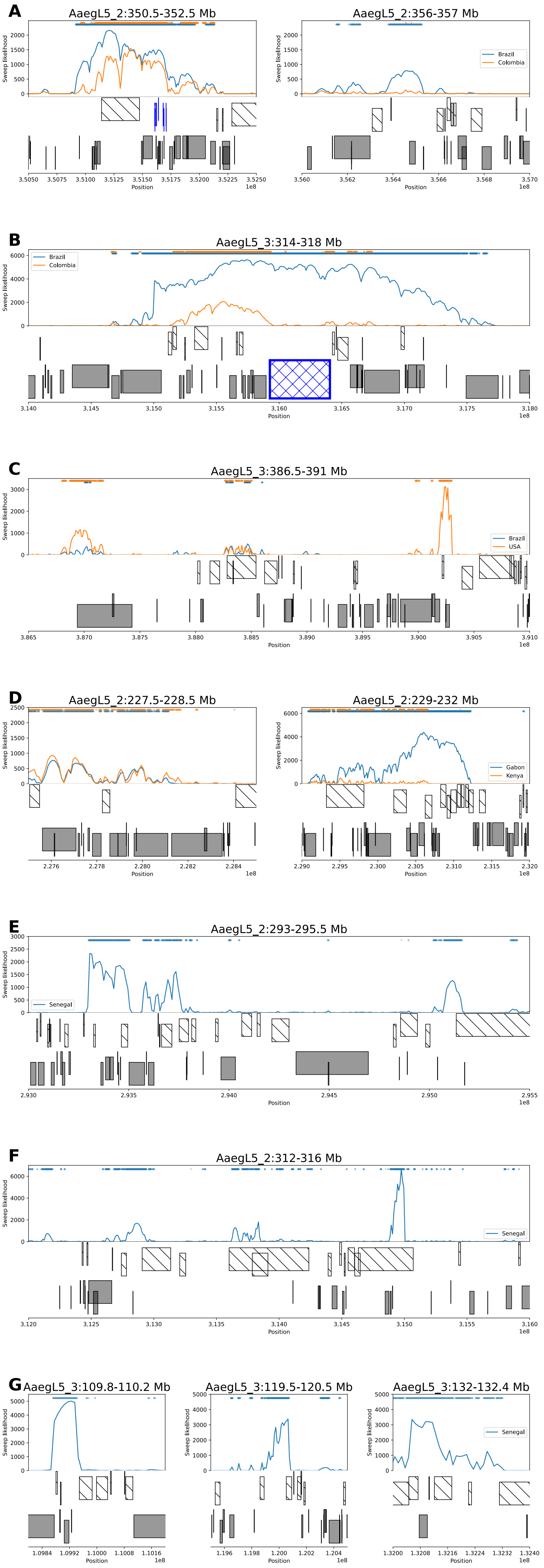

A
AaegL5_2:350.5-352.5 Mb
AaegL5_2:356-357 Mb
Sweep likelihood
Brazil
Colombia
Position
B
AaegL5_3:314-318 Mb
Brazil
Colombia
Sweep likelihood
Position
C
AaegL5_3:386.5-391 Mb
Brazil
USA
Sweep likelihood
Position
D
AaegL5_2:227.5-228.5 Mb
AaegL5_2:229-232 Mb
Gabon
Kenya
Sweep likelihood
Position
E
AaegL5_2:293-295.5 Mb
Senegal
Sweep likelihood
Position
F
AaegL5_2:312-316 Mb
Senegal
Sweep likelihood
Position
G
AaegL5_3:109.8-110.2 Mb
AaegL5_3:119.5-120.5 Mb
AaegL5_3:132-132.4 Mb
Senegal
Sweep likelihood
Position

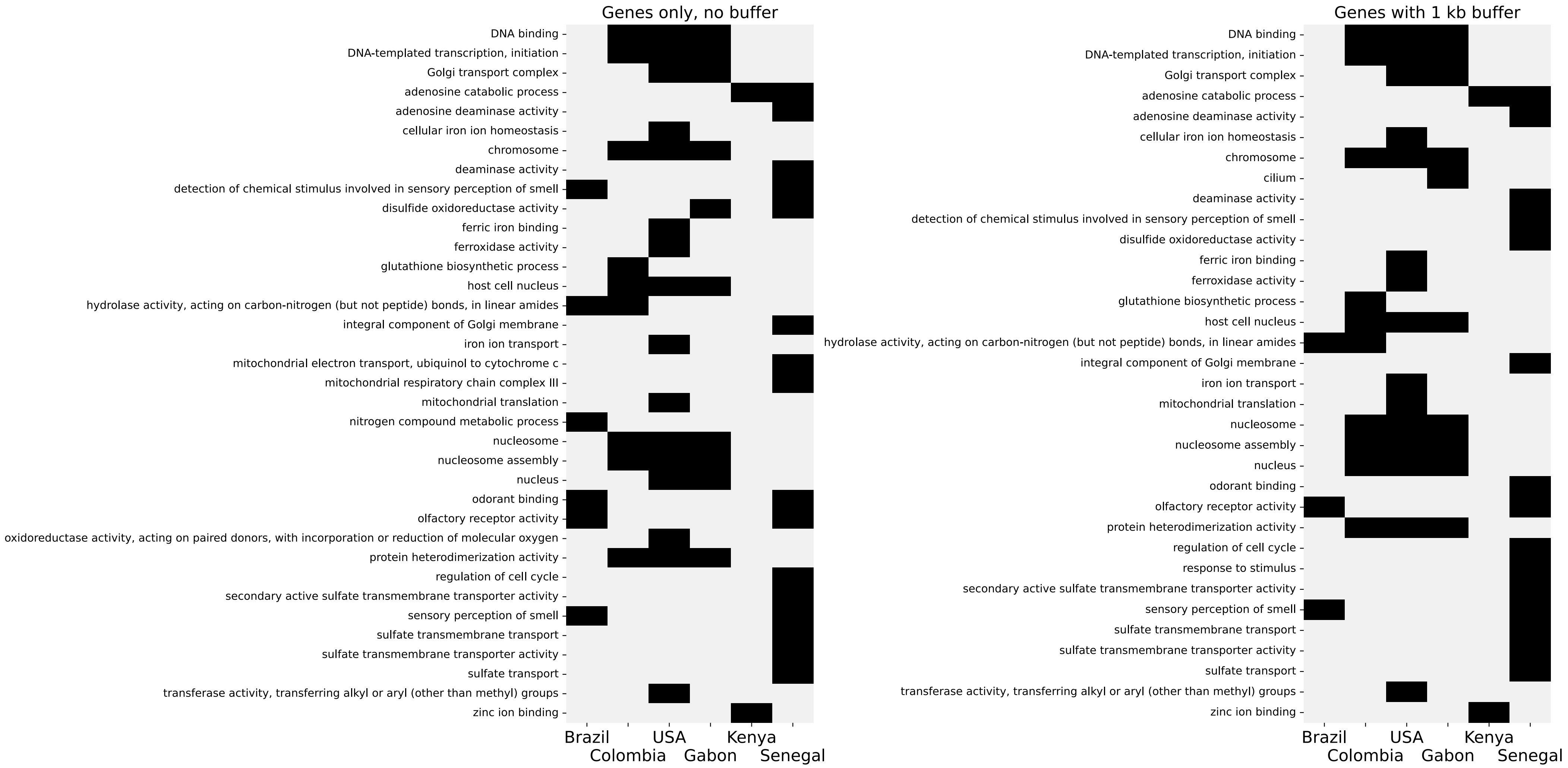
Genes only, no buffer
DNA binding
DNA-templated transcription, initiation
Golgi transport complex
adenosine catabolic process
adenosine deaminase activity
cellular iron ion homeostasis
chromosome
deaminase activity
detection of chemical stimulus involved in sensory perception of smell
disulfide oxidoreductase activity
ferric iron binding
ferroxidase activity
glutathione biosynthetic process
host cell nucleus
hydrolase activity, acting on carbon-nitrogen (but not peptide) bonds, in linear amides
integral component of Golgi membrane
iron ion transport
mitochondrial electron transport, ubiquinol to cytochrome c
mitochondrial respiratory chain complex III
mitochondrial translation
nitrogen compound metabolic process
nucleosome
nucleosome assembly
nucleus
odorant binding
olfactory receptor activity
oxidoreductase activity, acting on paired donors, with incorporation or reduction of molecular oxygen
protein heterodimerization activity
regulation of cell cycle
secondary active sulfate transmembrane transporter activity
sensory perception of smell
sulfate transmembrane transport
sulfate transmembrane transporter activity
sulfate transport
transferase activity, transferring alkyl or aryl (other than methyl) groups
zinc ion binding
Brazil
Colombia
USA
Gabon
Kenya
Senegal
Genes with 1 kb buffer
DNA binding
DNA-templated transcription, initiation
Golgi transport complex
adenosine catabolic process
adenosine deaminase activity
cellular iron ion homeostasis
chromosome
cilium
deaminase activity
detection of chemical stimulus involved in sensory perception of smell
disulfide oxidoreductase activity
ferric iron binding
ferroxidase activity
glutathione biosynthetic process
host cell nucleus
hydrolase activity, acting on carbon-nitrogen (but not peptide) bonds, in linear amides
integral component of Golgi membrane
iron ion transport
mitochondrial translation
nucleosome
nucleosome assembly
nucleus
odorant binding
olfactory receptor activity
protein heterodimerization activity
regulation of cell cycle
response to stimulus
secondary active sulfate transmembrane transporter activity
sensory perception of smell
sulfate transmembrane transport
sulfate transmembrane transporter activity
sulfate transport
transferase activity, transferring alkyl or aryl (other than methyl) groups
zinc ion binding
Brazil
Colombia
USA
Gabon
Kenya
Senegal

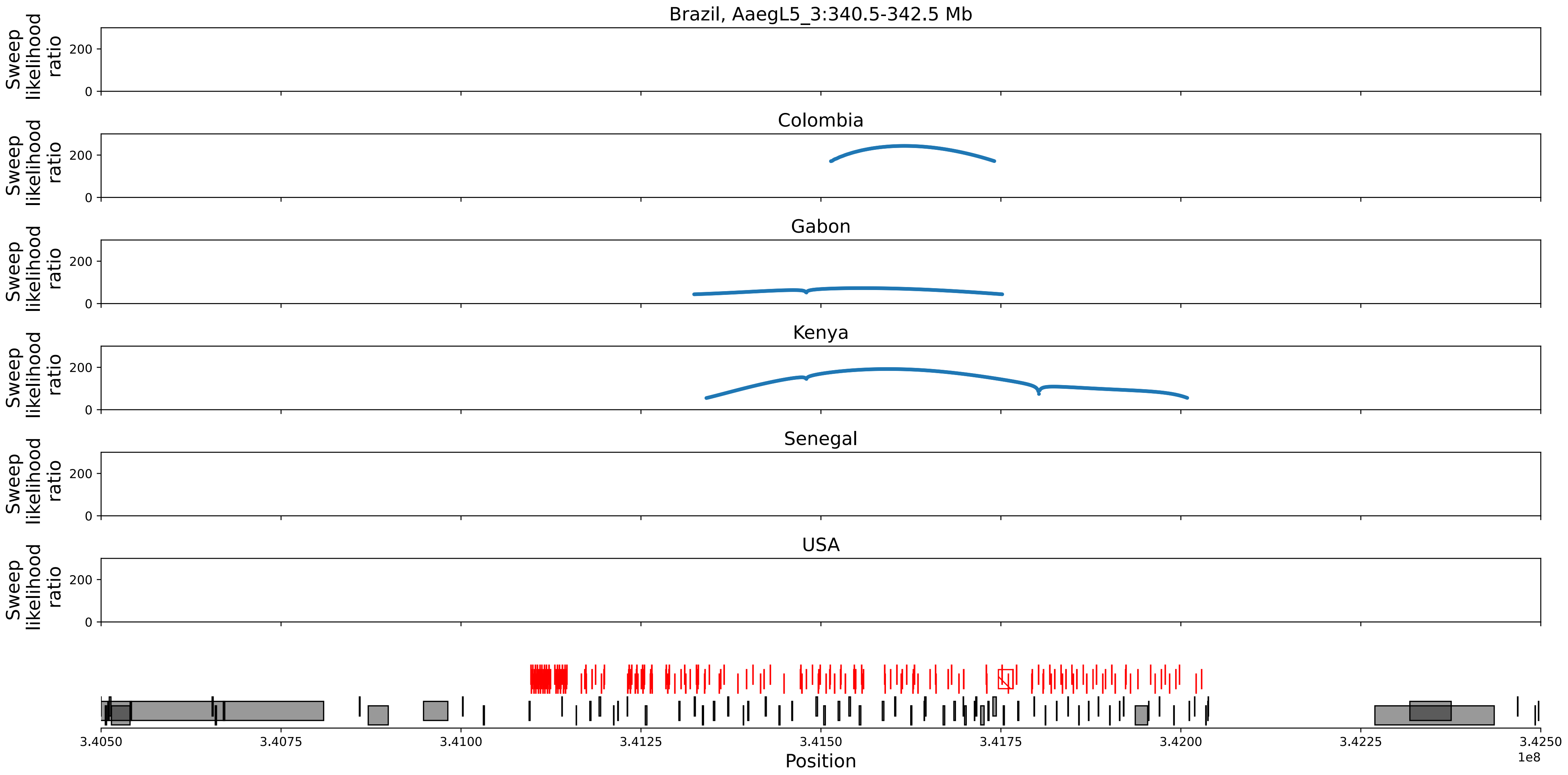

Brazil, AaegL5_3:340.5-342.5 Mb
Colombia
Gabon
Kenya
Senegal
USA
Sweep likelihood ratio
200
0
3.4050
3.4075
3.4100
3.4125
3.4150
3.4175
3.4200
3.4225
3.4250
1e8
Position

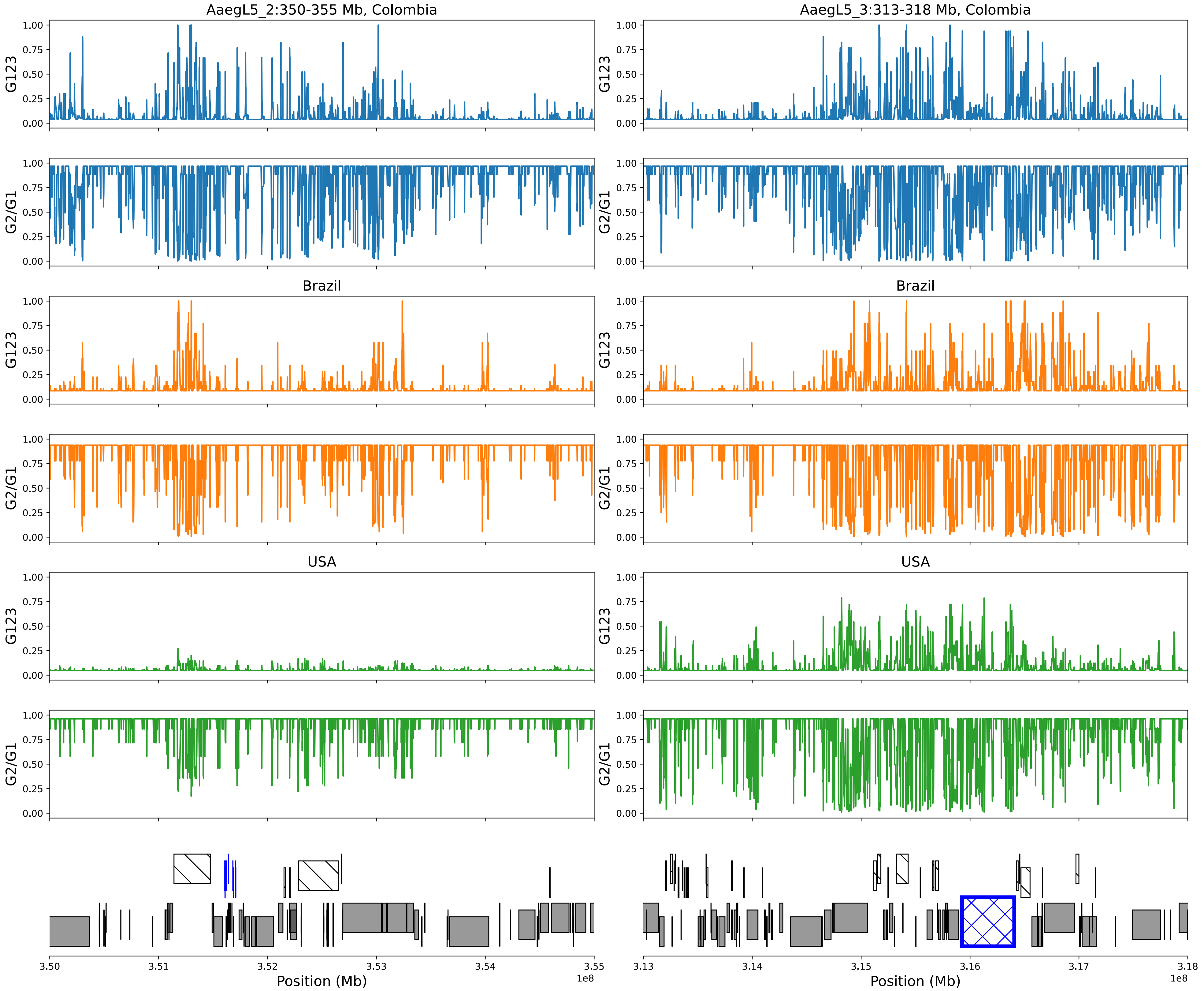
AaegL5_2:350-355 Mb, Colombia
AaegL5_3:313-318 Mb, Colombia
Brazil
USA
G123
G2/G1
1.00
0.75
0.50
0.25
0.00
3.50
3.51
3.52
3.53
3.54
3.55
1e8
3.13
3.14
3.15
3.16
3.17
3.18
Position (Mb)

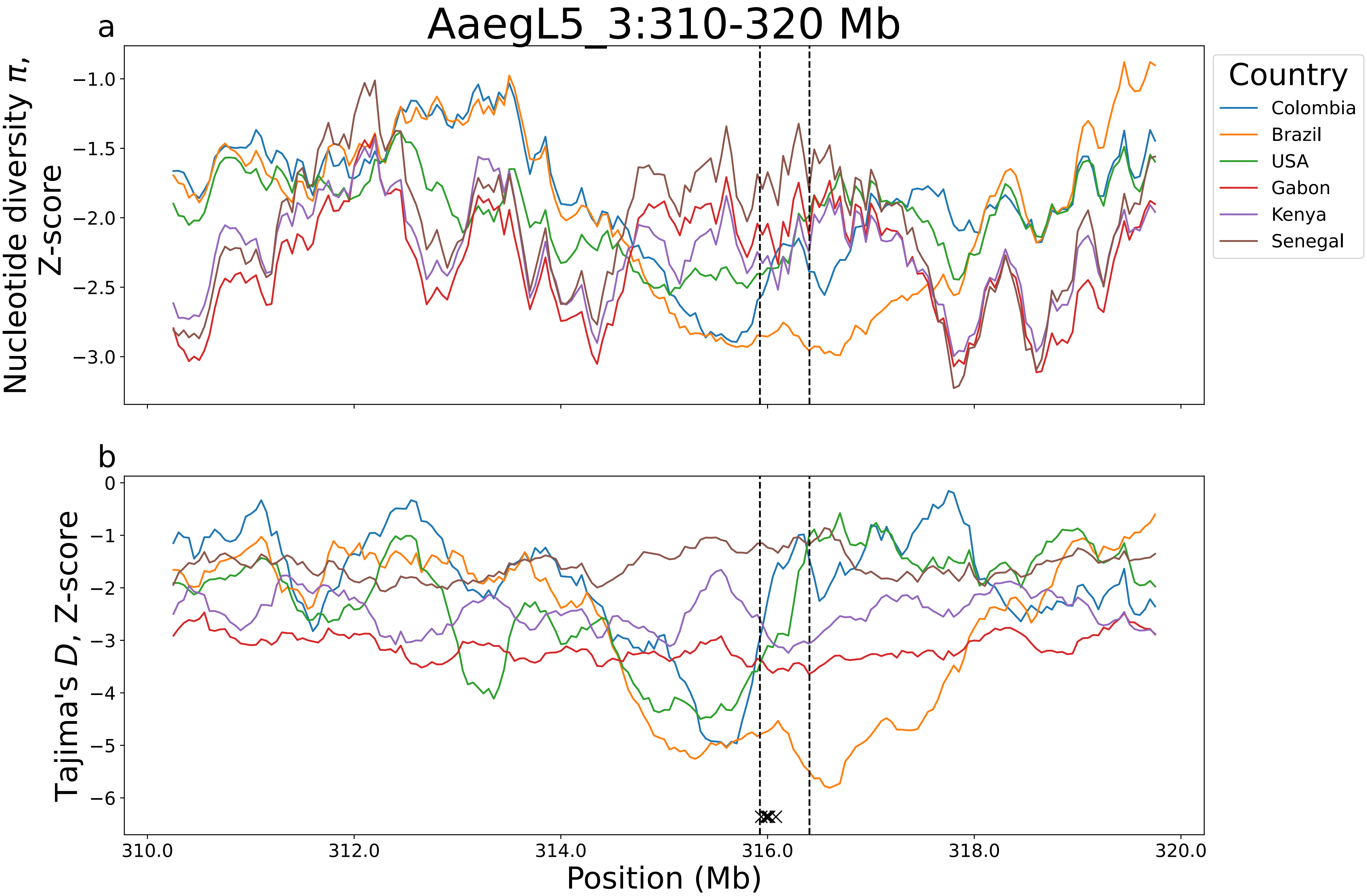
AaegL5_3:310-320 Mb
a
b
Nucleotide diversity π, Z-score
Tajima's D, Z-score
Position (Mb)
Country
Colombia
Brazil
USA
Gabon
Kenya
Senegal
−1.0
−1.5
−2.0
−2.5
−3.0
0
−1
−2
−3
−4
−5
−6
310.0
312.0
314.0
316.0
318.0
320.0

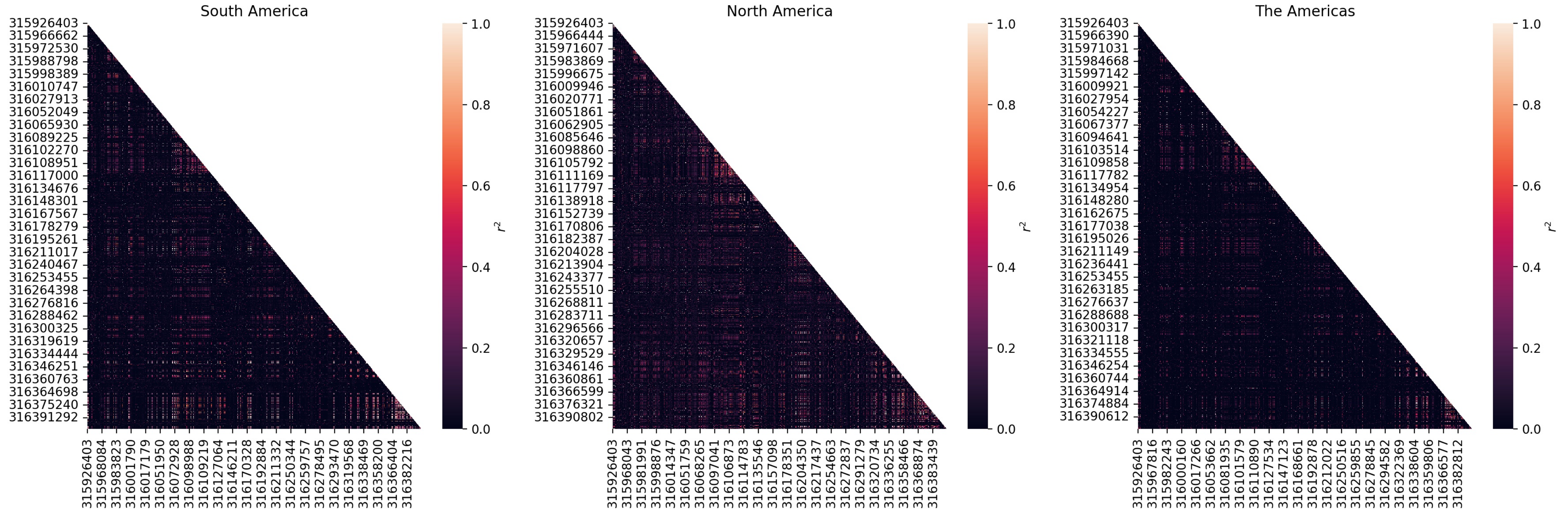
South America
North America
The Americas
1.0
0.8
0.6
0.4
0.2
0.0
r^2

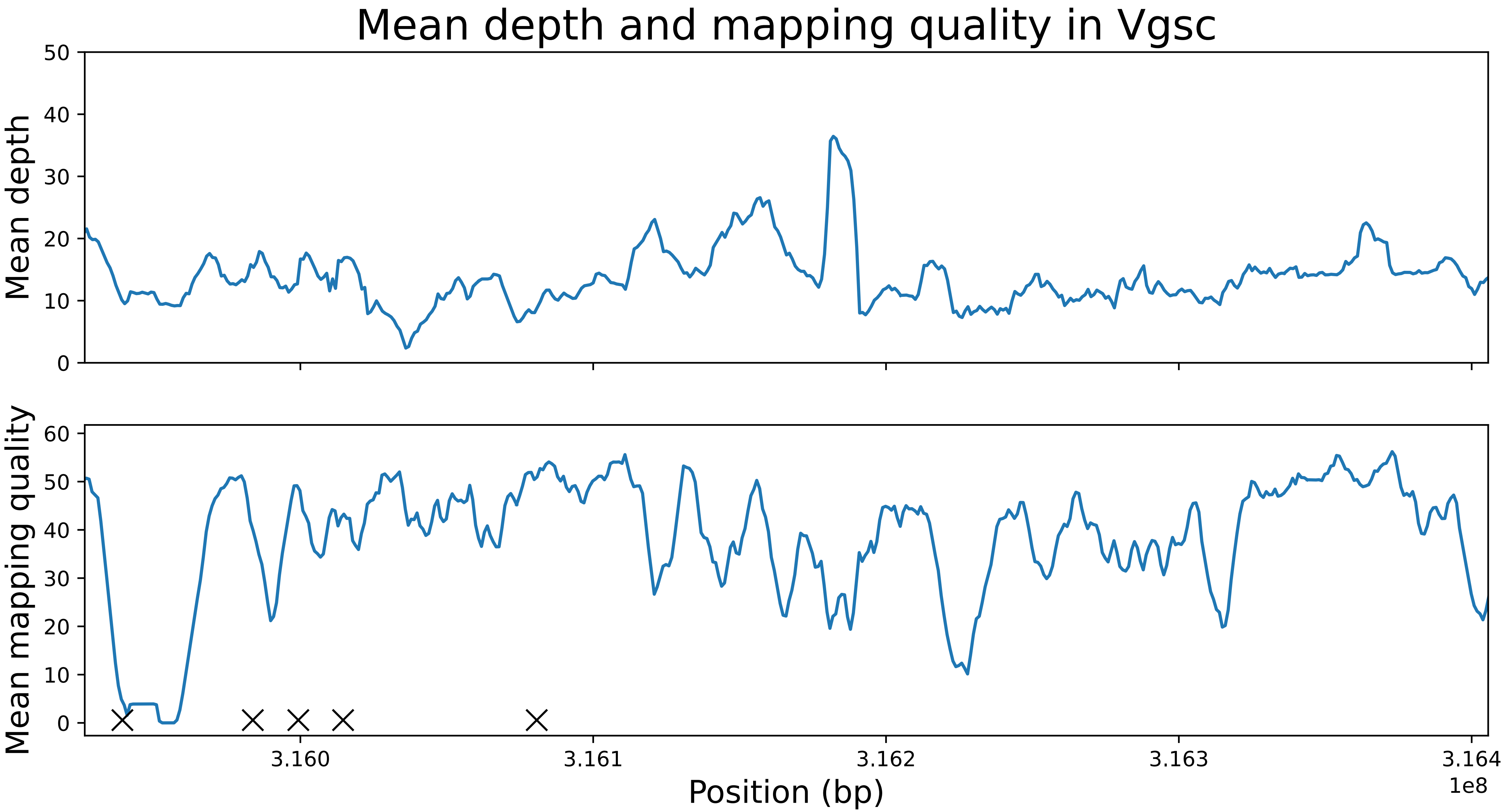

Mean depth and mapping quality in Vgsc
Mean depth
0
10
20
30
40
50
Mean mapping quality
0
10
20
30
40
50
60
3.160
3.161
3.162
3.163
3.164
1e8
Position (bp)

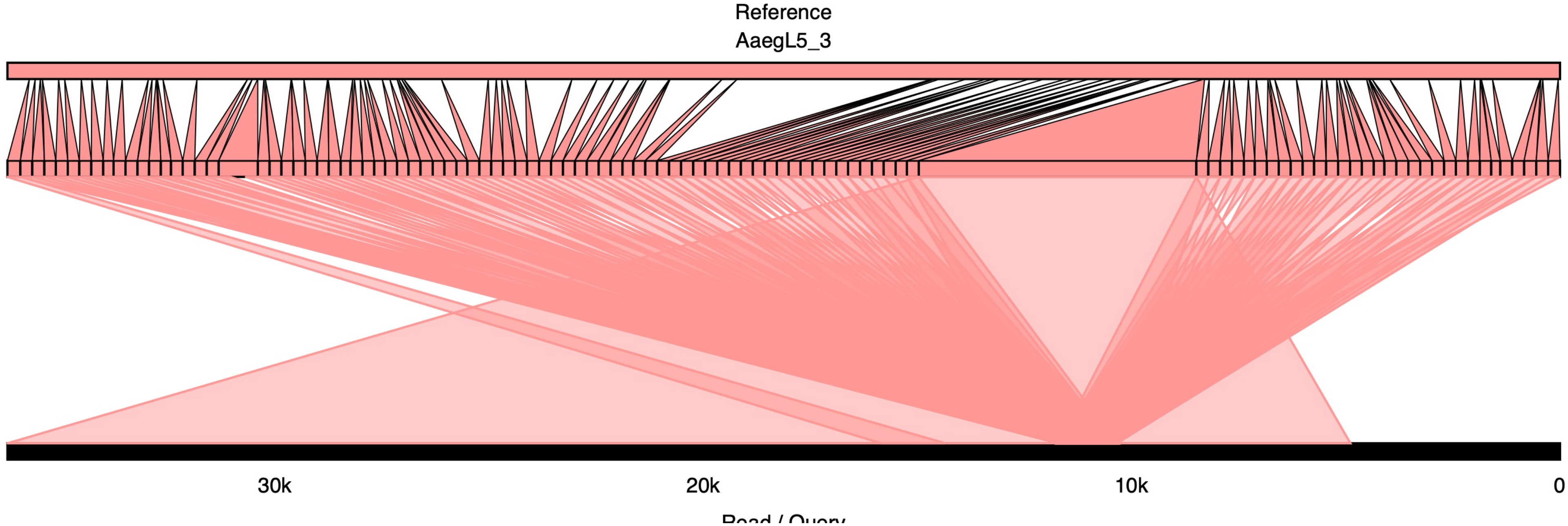
Reference
AaegL5_3
30k
20k
10k
0

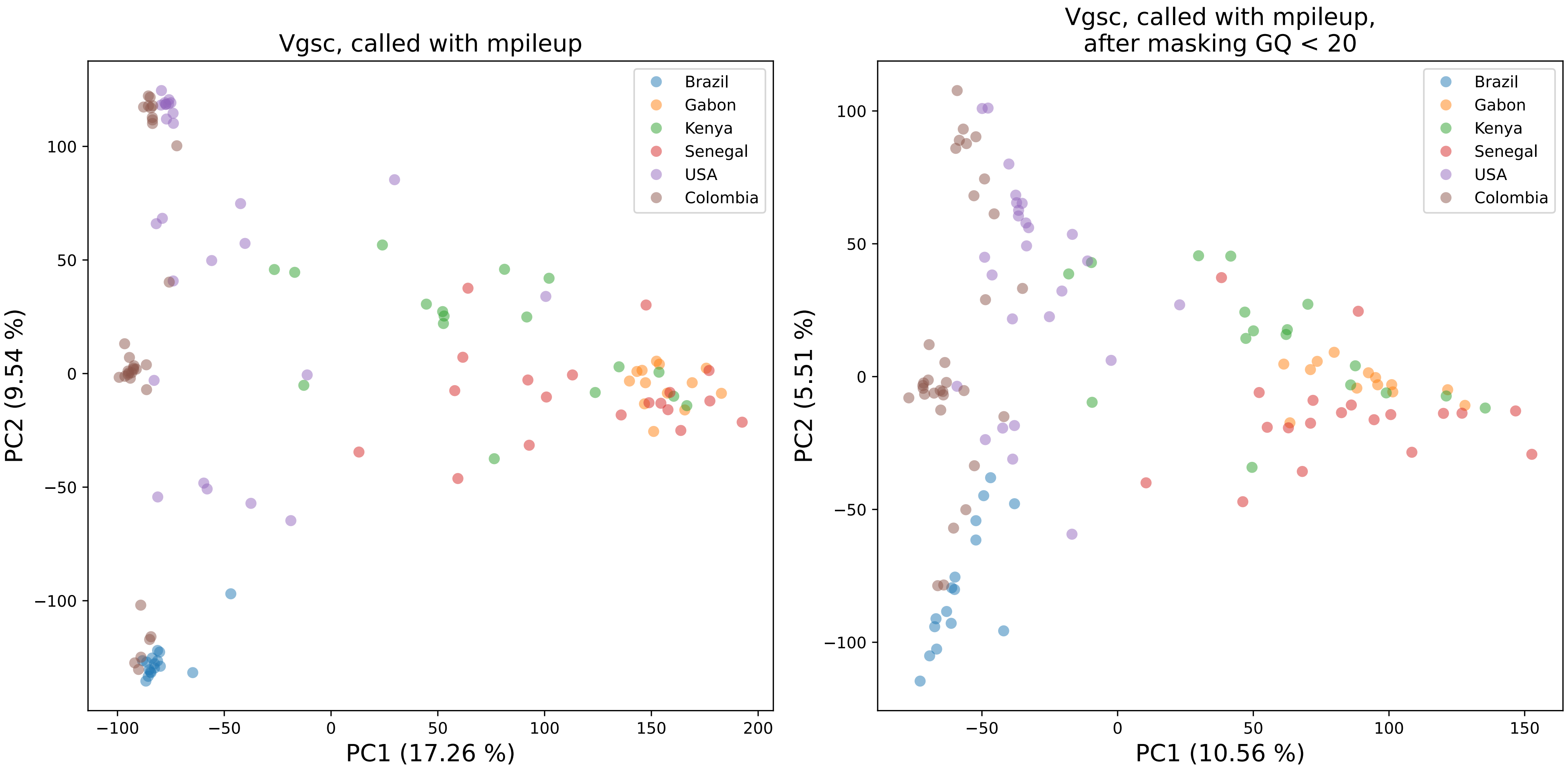
Vgsc, called with mpileup
Brazil
Gabon
Kenya
Senegal
USA
Colombia
PC2 (9.54 %)
PC1 (17.26 %)
Vgsc, called with mpileup, after masking GQ < 20
PC2 (5.51 %)
PC1 (10.56 %)

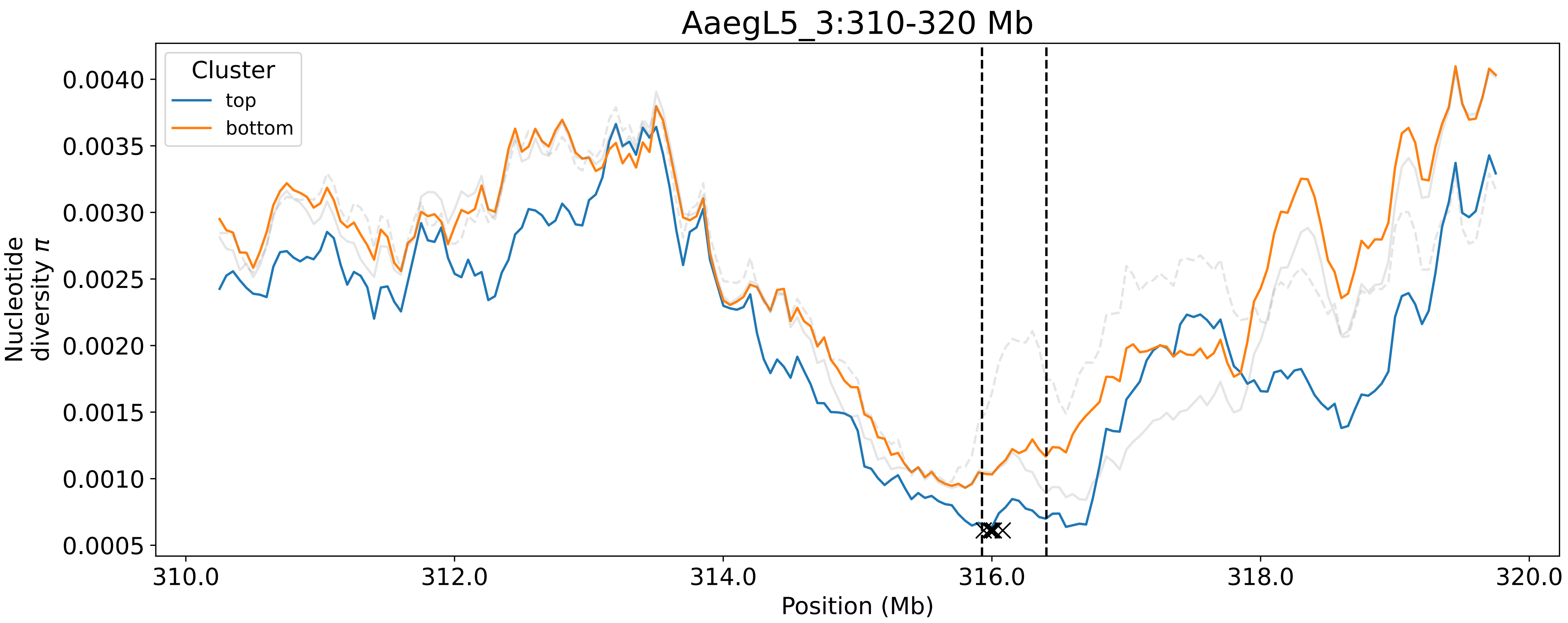

AaegL5_3:310-320 Mb
Cluster
top
bottom
Nucleotide diversity π
0.0040
0.0035
0.0030
0.0025
0.0020
0.0015
0.0010
0.0005
310.0
312.0
314.0
316.0
318.0
320.0
Position (Mb)

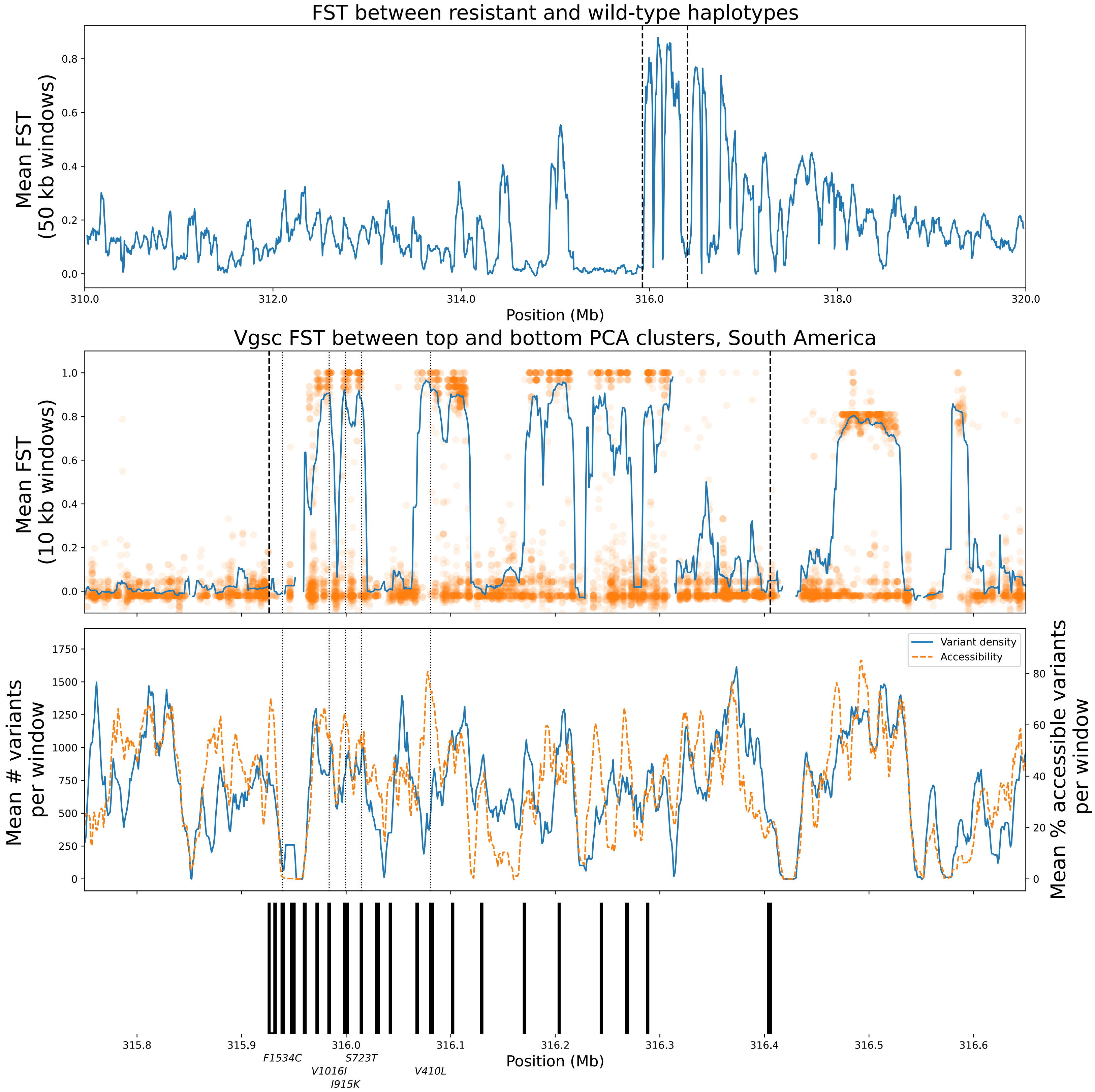
FST between resistant and wild-type haplotypes
Mean FST
(50 kb windows)
Position (Mb)
Vgsc FST between top and bottom PCA clusters, South America
Mean FST
(10 kb windows)
Mean # variants
per window
Mean % accessible variants
per window
Variant density
Accessibility
F1534C
V1016I
I915K
S723T
V410L
Position (Mb)

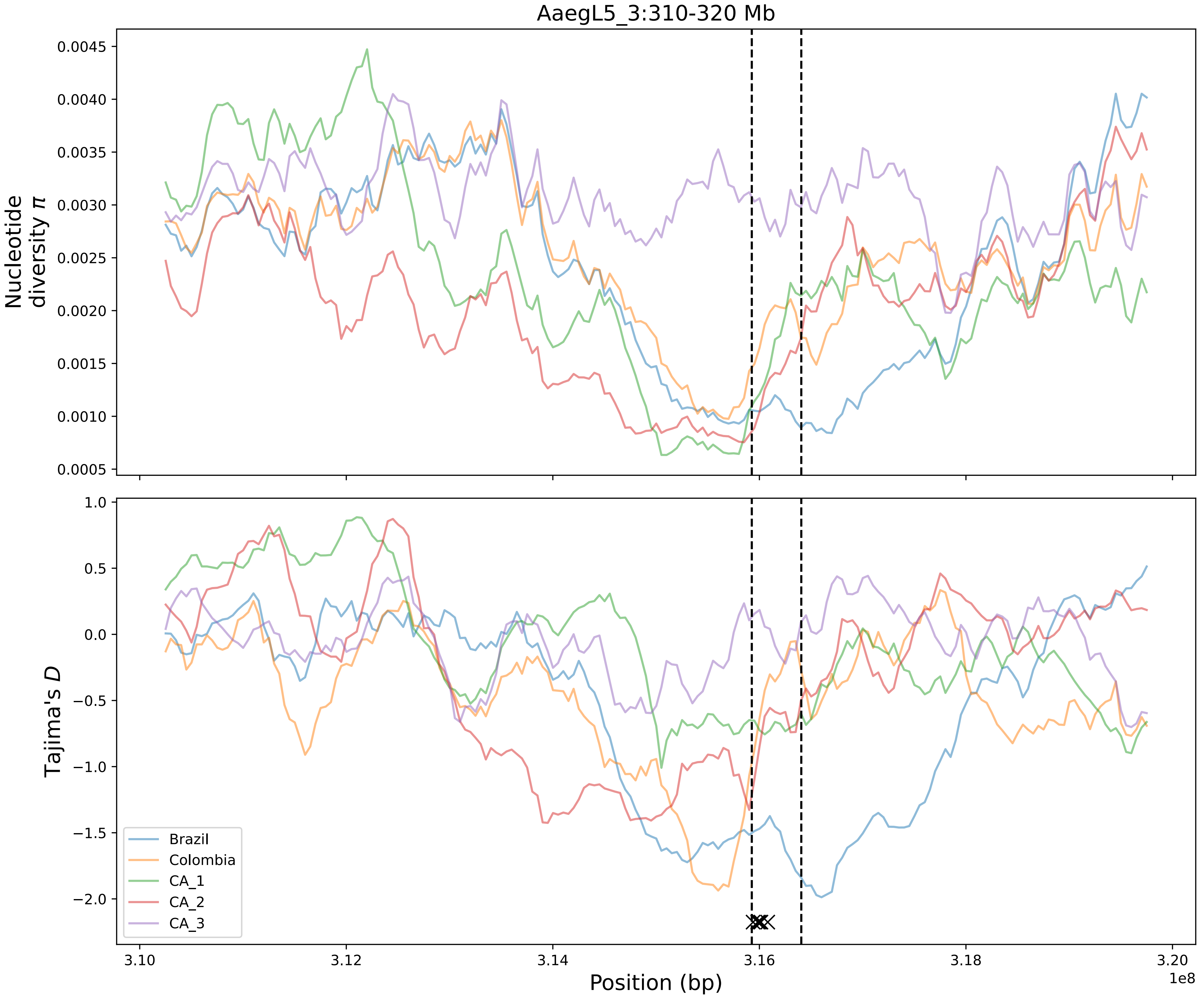

AaegL5_3:310-320 Mb
Nucleotide diversity π
0.0045
0.0040
0.0035
0.0030
0.0025
0.0020
0.0015
0.0010
0.0005
Tajima's D
1.0
0.5
0.0
−0.5
−1.0
−1.5
−2.0
Brazil
Colombia
CA_1
CA_2
CA_3
3.10
3.12
3.14
3.16
3.18
3.20
1e8
Position (bp)
